# University students’ help seeking intention for depression from health professionals; a cross sectional study

**DOI:** 10.1371/journal.pone.0271392

**Published:** 2022-07-13

**Authors:** Alemu Lemma, Woredaw Minichil, Endalamaw Salelew, Jinenus Tadesa, Habtamu Kerebih, Kabtamu Nigussie, Demeke Demilew, Shegaye Shumet

**Affiliations:** 1 Department of Psychiatry, School of Medicine, College of Medicine and Health Science, University of Gondar, Gondar, Ethiopia; 2 Department of Psychiatry, School of Nursing and Midwifery, College of Health and Medical Sciences, Haramaya University, Harar, Ethiopia; South African Medical Research Council, SOUTH AFRICA

## Abstract

**Objectives:**

To assess University students’ intention to seek help for depression from health professional and associated factors among University of Gondar students, Northwest, Ethiopia.

**Methods:**

An institution-based cross-sectional study was conducted among 487 students. Multistage sampling technique was used to select study participants. Self-administered questionnaire was employed to collect the data. General help seeking questionnaire with major depressive disorder case vignette was used to assess students’ intention to seek help for depression. The collected data was analyzed using SPSS version 20. Simple and multiple linear regression analysis were performed to identify factors for intention to seek help for depression. Association was described by using adjusted unstandardized β coefficient along with 95% confidence interval. Finally, P-values < 0.05 in adjusted analysis were taken as a cut off for significant association.

**Results:**

The mean score of intention to seek help from health professionals was 3.84 (±0.76)with a range of (1 “very unlikely” to 5 “very likely”). About 67.8% of the study participants would seek help from health professionals if they would have depressive symptoms which was depicted in the case vignette. In the multiple linear regression analyses, student age (unstandardized ß = 0.07, 95% CI: (0.05, 0.10)), good attitude towards seeking professional help (ß = 0.03, 95% CI: (0.02, 0.04)) were factors positively associated with intention to seek help.

**Conclusion:**

The current study showed that more than three-fifth of the study participants reported they were likely or very likely to seek help for depression from health professionals. Increased age and favorable attitude were factors associated with intention to seek help for depression. Working on awareness creation and attitude change towards depression would be necessary to increase students’ intention to seek help for depression from health professional.

## Introduction

Depression is a common mental health problem that presents with discouraged mood, loss of joy, diminished energy, low self-esteem, poor concentration and change of appetite [1]. According to the recent world mental health survey in 17 countries, 1 out of 20 young individuals have depressive episode [2].

Depressive symptoms are common in university students globally. A meta-analysis study among china university students revealed that the overall prevalence of depression was 23.8% [3]. Study results in African countries showed that the prevalence of screnned depression among university students ranges from 32-2-41.33% [4–6]. In Ethiopia, the prevalence of current depressive symptoms among University students was 32.2% [7]. Depression was found to cause a large portion of a latter grade decrease in students GPA, students complained about increased interference of depressive symptoms with academic tasks, leading to poor score on test and drop out from university [8]. Some study reported that depression contributes 7% of disease burden in Ethiopia [9].

Previous studies reported that age, transition from high school to university, thought of fewer job prospects after graduation, low levels of exercise and substance misuse were risk factors associated with depressive symptoms among university students [10–12].

Although 32.5% of the university students screened positive for major depression, which was indicative for health professional help, they are very reluctant to seek health professional care. As studies showed only few students sought help from any kind of help sources for their problem [13–15]. Study results in different university students indicated that most students use as help sources their family and parents for their mental health problems). Therefore, understanding intention of students to seek help is an important issue for early intervention of the burden of depression since intention is a predictor ofhelp-seeking behavior [16, 17].

From mental health point of view, help-seeking intention is an adaptive coping process that is an attempt to get external help to deal with a mental health concern Some studies revealed that the university students intention to help for depressive syptoms was low which is, 1.32–3.72% [18]. However, another studies showed. However, another studies showed that the students intention to seek psychologist professional help for depressive symptom was high, which is ranging from1.7–7.3%. A study conducted in Queensland university of Technology using online data collection the mean intention score was running between 4.34 and 9.72. Another study using a case vignette of depression and general help seeking questionnaire in Sri-lanka among university students reported as student help seeking intention from psychiatrist was 7.3% this indicated that only few students would seek professional help if they would encounter depression as depicted in the vignette [19]. High Intention to seek help for depression among Botswana university students was reported with a mean value ranging from 2.73 to 4.27 [20]. A cross sectional study in Ethiopia using major depressive case vignette with GHSQ revealed 2.49–4.95% of students intended to seek treatment [21].

There are many factors which predict intention to seek help such as sex [13, 22, 23], age [13, 24], capacity to recognize psychological problems [13, 25]. Family history of depression [18, 24], fear of addressing their problem and expectation of negative reaction from the experts [26, 27] and students’ social support [28] are another factors which influence intention to seek help.

Though depression is common among university students, they are reluctant to seek help from health professionals. Students with different mental health problems often seek help from religious and traditional healers rather than health facilities [9]. As a result, understanding intention of students to seek help for depressive symptoms is crucial to intervene the burden of depression because intention is a predictor of a behavior [29]. Litle data is knowing about the students intention to seek help for depressive symptom from health professional in eastern Africa as well as Ethiopia among university students which is become the main problems for intervention to the impact of depression. Therefore, the aims of this study was assessing students’ intention to seek help for depressive symptoms among University students in Ethiopia.

## Materials and methods

### Study settings

The study was conducted at the University of Gondar period from February to March 2020. University of Gondar is one of the oldest universities in Ethiopia. University of Gondar was established in 1954 as a Public Health College and training center. It has six colleges among that, University of Gondar College of medicine and health science currently offers about 12 undergraduate degrees for 3,455 regular students and College of Business and Economics offers 9 undergraduate programs for 1,530 regular students. The two college accounts 4,985 undergraduate students.

### Study design and population

Institutional based cross-sectional study design was conductedamong all undergraute university students aged 18 years and above, who had been available during data collection. Students who were unable to communicate, with serious health condition and on field work during data collection period were excluded.

### Sample size determinant and sampling technique

Adequate number of samples required for the study was determined by using (*δ* = 0.77) taken from Botswana study [20] and margin of error = 0.1 at 95% confidence level. With design effect of two and adding10% non-response rate the final sample size was (n = 502). A multi-stage sampling technique was employed, which involved two stages of a random selection of participants. The first stage was formed at the university level using colleges as clusters. The total sample size for the study was distributed proportionally across departments according to the number of students in each department. Finally, simple random sampling method (computer generated) was used in each selected department to select study participants.

### Data collection tools

Data was collected using self-adminstred structured, semi-structured questionnaire. The questionnaire contains four parts which are socio-economic characteristics such as sex, religious, marital status, ethnicity, filed of study, year of study residentilaity, clinical factors family history of mental illness, percied severity of depression, perceived cause of depression, previous histry of help seeking, psychosocial factors like social support, attitude towards seeking help and perceptionand and substance related factors. Outcome varible, Intention to seek help for depression was measured using general help seeking questionnaire (GHSQ) with major depressive disorder case vignettes. GHSQ was developed by Rickwood et al. [30] and it measures future help seeking intention by listing a potential help sources. Participants would then indicate how likely is the participant would seek help from the listed help sources. The tool has 7 point likert scale ranging from “1” extremely unlikely to”7” extremely likely. In our study we used 5 point likert scale “1”extremely unlikely to “5” extremely likely, which was adopted from previous study in Ethiopia, with a good internal consistency α = 0.87 [21]. In the current study, the internal consistency for intention to seek help for depression was α = 0.81. Participants were asked how likely they would seek help from health professionals if they feel depressive symptoms depicted in the case vignette. High scores for professional help showed a student had a good intention.

Attitude towards seeking professional help was assessed using attitude towards seeking professional psychological help (ATSPPH). ATSPPH is ten-items tool with a likert scale response which ranges from ‘1’ strongly disagree to ‘5’ strongly agree, and if participants had a high score in the scale after the items summed, the participant would have favorable attitude to depressive symptoms [20].

Social support was assessed by using Oslo 3-items social support scale with scores ranging from 3 to 14: 3–8 = poor social support; 9–11 = intermediate social support; and 12–14 = strong social support [31]. To assess perceived severity of depression students were asked “How severe do you think is the illness presented in the case vignette?” and the response was mild, moderate, severe, and very severe. We assessed perceived need for treatment by asking: ‘Do you think the illness in the case vignette needs treatment?” and the response was “yes/no”. Family history of depression were assessed by: “Do you have a family member who had experienced a similar situation described in the case vignette?” the response was “yes/no”. To assess current substance use, respondents were asked: “Have you ever used any substance (none medical use) for the last three months?”. Previous history of seeking help, If the respondent answers “YES” for the question “have you ever felt the way that described in the case vignette” Then another question will follow like “Did you sought help at that time”, if the respondent answers “YES” the respondent has previous history of help seeking.

Substance-related factors was assessed by Alcohol, Smoking and Substance Involvement Screening Test (ASSIST), which is a brief screening questionnaire developed and validated by the world health organization (WHO) to find out about people’s use of psychoactive substances was used to assess current and ever substance use history of the subject [32].

### Data collection methods

Data were collected through self-administered Amharic version a structured and sem-structured questionnaires. The data collection was facilitated by four trained bachelors of science in psychiatry nurses. In order to reach specific slected study participants using class respresentative from each section was contacted. The questionnaire was translated to the Amharic language to be understandable by all participants and re-translated back to English to ensure its consistency. One day training was given to data Facilitator about ethical principle, confidentiality and data management. Pretest was done on 5% of the final sample size at the Bahir Dar university. The result was not included in the result of this study finding. Based on the finding of the pretest data, the questionnaire was checked for its clarity, simplicity, and understandability. The data collectors were supervised daily, and the field questionnaires were checked daily for completeness by the supervisors and the principal investigator.

### Data processing and analysis

The data was checked for completeness, consistency and entered into Epi-Data version 3.1 and then exported to SPSS (Statistical Package for Social Science) version 20 for analysis. Descriptive and simple linear regression analyses were computed to see the frequency distribution and to test the association between independent and dependent variables, respectively. Variables associated with intention to seek help for depression were selected during the simple linear regression analysis with a p-value < 0.2 for further multiple linear regression analysis. In multivariable regression variables with P<0.05 at a 95% confidence interval was considered as statistically significant. Goodness of model fitness test (R^2^) was checked and R^2^ was 0.582.

### Ethical considerations

Ethical clearance was obtained from the Ethical Review Committee of the University of Gondar, College of Medicine, and Health science. Formal letter of permission was obtained from the Department of Psychiatry and submitted to college of medicine and health science and the College of Business and Economics. Prior to data collection, both verbal and written informed consent was obtained from participants. The right to participate, to refuse or discontinue participation at any time they want and the chance to ask any thing about the study was given for the participants. Participants were informed about the aim of the study, procedures of selection, and assurance of confidentiality, and their names were not registered to minimize social desirability bias and enhance anonymity.

## Results

Out a total of 502 sample, 487participants were included in the study with a response rate of 97.0. The mean and standard division (SD) age of the participants was 21.64 and ± 1.55 years respectively. More than half 52.2% (254) of the participants were male. The age of the study participants was ranging from 18–29 years. Nearly two- third, 65.9% (321) were Orthodox Christian religious followers. The majority of the participants, 89.9% (438) were single in marital status and Urban residential was accounted for around 56.7% (276) as show below in (Table 1).

**Table 1:** Socio-demographic characterstics of undergraduate students of university of Gonder, northern part Ethiopia (n = 487).

Varibles	Categories	Frequency	Percentage (%)
Age	Mean	21.65	1.56
Sex	Male	254	52.2
	Female	233	47.8
Religion	Orthodox	321	65.9
	Muslim	75	15.4
	Protestant	74	15.2
	Catholic	13	12.7
	Others	4	0.8
Marital status	Single	438	89.9
	Married	38	7.8
	Divorced	11	2.3
Ethnicity	Amhara	259	53.2
	Oromo	94	19.3
	Tigre	25	5.1
	SNNP	102	21.5
	Others*	7	1.4
Field of study	Business and Economics	194	39.8
	Medicine and health science	293	60.2
Place of Residence	Rural	211	43.3
	Urban	276	56.7

Others* Somali, anyuwak.

### Clinical, psychosocial and substance related factors of the participants

About more than half 57.9% (282) of study participants had history of ≥1 depressive symptoms, 56(11.5%) of the participants sought help from health professionals for their depressive symptoms. Nearly, one-fourths (24.0%) of the study participants had family history of ≥1 similar symptoms. Regarding perceived severity of depression, 38.4% (187) of the participants perceived depression as severe. Out of a total participants more than half of them, 57.9% (282) had poor social support. In respect perceived causes for depression showed that, 71.4% (361) of the respondents perceived economic problems. About current substance use more than half of respondents, 59.8% (291) were used one or more type of substances and among those current Alcohol use account around half, 53.2% (259) of them as shown below in (Table 2).

**Table 2:** Description of clinical, psychosocial and substance use related features of undergraduate students of university of Gonder, northern part Ethiopia (n = 487).

Variable	Categories	Frequency(n)	Percentage (%)
Perceived severity of depression	Mild	70	14.4
	Moderate	154	31.6
	Severes	187	38.4
	very severe	76	15.6
Social support	Poor	282	57.9
	Moderate	134	27.5
	Strong	71	14.6
Perceived cause of depression			
Psychosocial	Economic problem	361	74.1
	Class load	318	65.3
	Loss of loved ones	343	70.4
	Excessive worrying	352	72.24
	Substance use	270	55.4
	Conflict with family relatives	250	51.3
Physical	Brain injury	203	41.7
	Neurochemical imbalance	232	47.6
Spiritual	Evil-spirits	174	35.7
	Buda-kalicha	129	26.5
	Punishment by God	143	29.4
Current alcohol use	Yes	259	53.2
	No	228	46.8
Current Khat use	Yes	96	19.7
	No	391	80.3
Current cigarette use	Yes	97	19.9
	No	390	80.1
Life time substance use	Yes	280	57.5
	No	207	42.5

### Intention to seek help for depression from health professionals

The overall intention to seek help for depression from any health professional was 67.8% with mean score of 3.84 (SD = 0.76). Nearly half of them 48.7% reported as they would “likely” and 19.1% would “very likely” aimed to visit health care providers for their illness delineated in the vignette suggesting that most of the respondents had an aim to visit health professionals.

### Factors associated with intention to seek help for depression

In the simple linear regression analysis variables; age, poor and moderate social support, field of study, participants’ history of depressive symptoms, perceived severity of depression, attitude, past history of help sought and perceived need for treatment were candidate for multiple linear regression analysis with a p-value less than 0.2. In the multiple linear regressionanalysis only variables age, poor social support, moderate social support, field of study, attitude, perceived need for treatment, moderate perceived severity and mild perceived severity were significantly associated with intention to seek help for depression at p value < 0.05.

As age increase by a year help seeking intention for depression from health profession increase by 0.075(ß = 0.075, 95% CI (0.045, 0.104)). The score of intention to seek help for depression increased by 0.033 (ß = 0.033, 95% CI (0.024, 0.043)) for every unit increase in attitude towards seeking professional help.

The value of intention to seek help for depression from any health profession for depression decreased by 0.112 (ß = —0.112, 95% CI (-0.205, -0.019)) for every student who attend business and economics class. The score of intention to seek help from health professional turned down by 0.342 (ß = -0.342, 95% CI (-0.443, -0.240) for every student who perceived as no treatment is needed for depression. For students who had poor social support, intention to seek help for depression from any health professional diminished by 0.748 (ß = -0.748, 95% CI (-0.896, -0.600)). Intention to seek help from health professional reduced by 0.557 (ß = -0.557, 95% CI (-0.734, -0.380)) and 0.201 (ß = -0.201, 95% CI (-0.348, -0.054)) for every student who perceived depression as mild and moderate respectively as shown below in (Table 3).

**Table 3:** Factors associated with help seeking intention for depression from health professionals. Simple and multiple linear regression analysis among University of Gondar students (n = 487).

Variables	Crude ß (95% CI)	adjusted ß(95% CI)
Constant	-	2.171 (1.460, 2.883)
Age	0.158 (0.116–199)	0.075(0.045, 0.104)***
Field of study		
Health science	0	0
Business and Economics	-0.210 (-0.348, -0.073)	-0.112(-0.205, -0.019) *
Perceived severity		
Very severe	0	0
severe	0.272 (0.134, 0.410)	0.002 (-0.138, 0.143)
Moderate	-0.238 (-0.382, -0.093)	-0.201 (-0.348, -0.054) **
Mild	-0.812 (-0.991, -0.632)	-0.557 (-0.734, -0.380) ***
Participants’ hx of depressive symptoms	-0.124(-0.20, -0.001)	-0.100 (-0.261, 0.013)
Social support		
Strong social support	0	0
Moderate social support	0.008 (-0.144, 0.160)	-0.687 (-0.839, -0.530) ***
Poor social support	-0.600 (-0.727, -0.474)	-0.748 (-0.896, -0.600) ***
Past history of help sought	-0.074(-0.07, 0.04)	-0.003(-0.149, 0.153)
Attitude	0.074 (0.063, 0.084)	0.033 (0.024, 0.043) ***
Perceived need for treatment		
Yes	0	0
No	-0.714 (-0.842, -0.587)	-0.342 (-0.443, -0.240)***

*P- value < 0.05,

** *P*-value < 0.01, and

***P-value < 0.001, VIF = 1.08–2.80, R^2^ = 0.582.

## Discussion

This study finding show that significant number, nearly one third of students intended not to seek any health professional help. This might be because of university students intend to overcome emotional problems alone or seek help on internet [33]. Other reason might be because of youngsters enjoy seeking help from informal sources such as friends and close relatives rather than health professionals [34]. Our finding on this study is consistent with the previous study at Australia University [27]. It’s also in line with a cross-sectional study on Turkey university students [22].

The current finding is also in line with study conducted in Queensland University using the same tool but different range 0 (extremely unlikely) to 7 (extremely likely), were majority of students reported to have good help seeking intention from health professional [35]. The finding of the current study is also similar with the study conducted in Botswana university students, where greater portion of the students reported as they had good intention to seek professional help for depression [20]. This finding is consistent with study done at northwest of Ethiopia using the same tool [21] but the study done in northwest was a community-based study which was composed of diversified participants in terms of socio demography whereas the current study included only univesrsity students.

However, the current finding was lower as compared to another community based study conducted in Mertule-mariam northwest and jimma southwest Ethiopia [36, 37]. As a matter of fact, socio-demographic difference between the study participants in which only university students aged 18 to 29, participated in our study while, people of any age and large sample size was used in Mertule-mariam’s study might be the reason for the difference and study done in jimma university study not only about depression it is done among the whole common mental disorders. Other possible difference might be due to age of study paricipants which is study done at Mertule-mariam’s of nearly two third of participants were aged 30 years and older, as help seeking intention increased with increasing age and tools used to assess the outcome varibles which is the study done in jimma university us using the Actual Help-Seeking Questionnaire [37]. Regarding associated factors, age was significantly associated with intention to seek help for depression from any other health professionals. The result is consistent with the study at Turkey, which study in turkey was reported that as age increase professional help seeking intention increases [22]. In study conducted in England being in older age was associated with better professional help seeking intentions [38]. Older adults have a positive attitude to seek professional help for mental illness [39]. Possible reasons for this might include, as age increment distinguishing, explaining and dealing with their feeling (emotional competence) might be increased [40]. The other possible reason might be as age increase the help seeking intention from health professional can be increased as compared with youngersters because the depression did not seen as minor among adult population [21].

Attitude was significantly associated with intention to seek help from health professionals’ in the current study. This result is supported by the study done in Botswana University [20]. Another cross sectional study revealed as good attitude were matched to higher professional help seeking [39]. The study on Asian and Asian American college students [41] indicatedas positive attitude of the students to depression treatment increases the tendency to seek help from mental health professionals, which is also in line with the study on Latino immigrants [42]. The more positive attitude of the respondents to depression care, intent to seek help from health professionals increased. It may be a direct result of individuals with a positive attitude about depression may disclose their disease to health professionals or positive convictions/beliefs that health professional is helpful which brings a positive attitude [21].

There was a negative association between mild and moderate perceived severity of depression and help seeking intention in our finding. This might be because of severe perceived severity increases intention to seek help [43]. It might also be because of they consider depression as normal phenomenon when it’s not severe enough.

Our study revealed that poor social support associated negatively with intention to seek help for depression from health professional, it implies that when poor social support increased helps seeking intention decreased this is consistent with the studies conducted in different parts of the world [21, 22, 36, 44]. This might be due to participants who have good social support had an increased intention to seek help for depression from health professionals and a close support increases attitude and intention to seek help [28, 45].

Participants who responded “no” to perceived need of treatment for depression associated negatively with intention to seek health professional help. This might be due to respondents considers depression as not severe and need to utilize other help sources than health professionals. This finding in line with a cross sectional study conducted in Ethiopia among community residents [21]. This is area to be intervened as neglecting to look for help or postponing the help seeking process can lead for unwanted health outcomes, for example, substance misuse, participating in dangerous sexual conduct, lower nature of grown-up life and sudden death [46].

There were also a negative association between being business and economics student and intention to seek help from health professionals. This might be because of low awareness about mental health and might be due to they consider depression as mild and normal phenomenon and the socio-cultural aspect of mental in Ethiopia since most of people consider tradional method of help seeking rather from health professional [47].

### Limitation of the study

One limitation, failed to identify those respondents whom have depression or not before we assessed their intention, as an intention for someone who had the symptoms and who hadn’t been, is not the same. Furthermore since the current study was cross sectional, which failed to assess the cause and effect relationship among the outcome variable and predictor variable.

## Conclusion

The intention to seek help for depression from health professionals’ was relatively low than general population. Increased age, positive attitude towards depression, and perceived need for treatment were factors positively associated with intention to seek health professional help. On the contrary, poor social support, perceiving depression as mild and moderate, no needing of treatment for depression, and being a business and economics student were factors negatively associated with intention to seek health professionals’ help. Students should be addressed in the importance of the need for treatment for mental health problems and University and other stakeholder should screeing and set plan for students with depression. To provide a strong attention to students those had poor social support, precived depression as mild and moderate and business and economics students while we concern about intention to seek help for depression from other health professional.

## Supporting information

S1 Data (SAV)

## List of abbreviation

ATSPPH: Attitude Towards Seeking Professional Psychological Help
CBE: College of Business and Economics
DSM: Diagnostic and Statistical Manual of Mental Disorders
GHSQ: General Help Seeking Questionnaire
GPA: Grade point average
SPSS: Statistical Package for Social Science
WHO: World Health Organization
